# Supplementary material for: Comparing the effectiveness and safety of Abatacept and Tocilizumab in elderly patients with rheumatoid arthritis
Source: PLoS One. 2022 Sep 19;17(9):e0274775. doi: 10.1371/journal.pone.0274775 (PMC9484651; doi:10.1371/journal.pone.0274775)
Supplement: Attachment — Submitted filename: Reviewer response new.docx [file pone.0274775.s002.docx]

Reviewer #1: The authors compared the efficacy and safety between
abatacept (ABT) and tocilizumab (TCZ) in elderly patients with
rheumatoid arthritis (RA). The focusing point of this study is important
and interesting in the era of ageing society. The authors concluded that
drug retention rates were equivalent between ABT and TCZ, but the rates
of discontinuation due to adverse event (AE) seemed to be lower with ABT
than TCZ in elderly patients with RA. However, this study contains
several serious concerns.

major
1. The authors should make a strict distinction between the terms of “
Elderly-onset RA” and “Elderly RA”. These two terms refer to different
population of RA (i.e the former refers to RA diagnosed at age of 65 or
older, and the latter refers to patients who are currently 65 years or
older regardless of the age of RA onset.) and which population should be
targeted depends on the aim of the study, however, the authors use these
terms confusingly. Therefore, the authors should reviewthe design of the
study and definition of patients depending on the aim of this study and
revise the manuscript.

We appreciate your critical comments. According to your important comments, we discriminate “Elderly RA” and EORA and corrected these descriptions in the revived manuscript. We also performed the same analysis using the selected elderly-onset RA patients as requested by Reviewer #2. We presented these new data in the revised manuscript.

2. The authors showed all AEs leading to the discontinuation of ABT or
TCZ in Table 2, but some AEs (ILD, liver dysfunction, renal dysfunction,
cardiovascular disease, hypothyroidism) are generally unlikely to be
associated with ABT or TCZ administration. Thus, it is questionable
whether those AEs are due to these drug administrations and
discontinuation of ABT or TCZ were necessary. Thus, the author should
add explanations about detailed situation of each AE.

We appreciate your critical comments. According to your important comments, we described the detailed situations concerting the discontinuation of TCZ or ABT due to AEs in the revised manuscript.

3. The authors highlighted the risk of ILD in the TCZ group, but it is
not well verified or described. First, it is necessary to describe
whether the ILDs were new occurrences after the initiation of TCZ or
exacerbations of already diagnosed. Also, effect of MTX should be
considered.

We appreciate your critical comments. According your comments, we described the detailed information concerning the occurrence of ILD and the effects of MTX in TCZ group in the revised manuscript.

4. Regarding efficacy, the authors only showed achieving rate of low
disease activity according to CDAI and DAS28-CRP at 24 weeks. When I read
the method (P7L2-5), it seems that this did not include remission. It is
desirable to analyze it including remission to evaluate effectiveness of
the treatments. The authors should also show the longitudinal changes of
score of CDAI and DAS28-CRP, proportion of the category of each disease
activity (remission, low-, moderate-, high-disease activity), each
component of composite measures, if possible.

We appreciate your critical comments. According to your precise comments, we presented these important data in the new Figure in the revised manuscript.

5. Please describe summary of rate of reasons for drug discontinuation
in ”Drug overall retention rates” section in P11.

According to your important comments, we described the rates of reasons for drug discontinuation in this section of the revised manuscript.

6. AEs should be listed as a whole AE and then divided into AEs that led
to drug discontinuation and AEs that did not.

According to your important comments, we listed whole AEs dividing into AEs leading to or not-leading to drug discontinuation in the revised Table 2.

7. The author excluded patients with no history of csDMARDs in Figure 1.
What is the reason for excluding these patients?

We appreciate your important comments.

These patients were excluded from the analysis due to the following reasons:

Since the pre-existing serious complications (progressing ILD, renal insufficiency, hematological disorders, advanced disability), which limit the use of MTX and may affect the clinical course in these patents.

8. The authors should add more detail clinical characteristics at the time of initiation of ABT or TCZ in Table 1. Each component of composited measures (SJC, TJC, PtGA, PGA), concomitant use of csDMARDs other than MTX, proportion of chronic kidney disease (eGFR<60), dose of glucocorticoid, types of used bDMARDs should be added.

We appreciated your critical comments. According to your comments, we presented this important information in new Table 1 in the revised manuscript.

9. Table 1 showed 12 patients with ABT group and 25 patients with TCZ group have ever used prior bDMARDs use. Is that mean are all prior bDMARDs TNF inhibitors? If not, it is necessary to describe the person who used both ABT and TCZ during the period.

We appreciate your important comments. According to your comment, we added these important information in revised Table1.

Minor
1. Please correct abbreviations in the manuscript and figures.
Abbreviations should be defined at first mention and used consistently thereafter, but it has not complied with frequently (ex. Adverse event (AE), low disease activity (LDA), abatacept (ABT, ABA) etc.).

We corrected these abbreviations in the revised manuscript.
2. “In the matched analysis,・・・・(P2L12, in abstract)” is not
appropriate sentence because it may mislead that background were
adjusted by using Propensity score matching etc.

We appreciate your critical comments. We deleted these sentences in the revised manuscript.

3. In P8-9, check and correct the results for the rate of glucocorticoid
use, as the results are opposite in the text and in Table 1.

We corrected these mistakes in the revised manuscript.

4. In Table 3, the number of MTX users in the AE group is incorrect.

We corrected these mistakes in the revised manuscript.

Reviewer #2: This study compared efficacy and safety of Abatacept and
Tocilizumab in elderly patients with rheumatoid arthritis and showed
comparable efficacy between both agents. On the other hand, the rates of
discontinuation due to AEs, including ILD, seem to be lower with ABT
than with TCZ in elderly patients with RA.

Major comments
The reviewer has a concern with the inclusion criteria.
#1 This study enrolled elderly (age ≥65 years) patients, irrespective of
onset age with a mean of 7 years. Onset age is one of the important
factors to determine clinical features including therapeutic responses.
The reviewer is afraid that the authors confuse clinical features of
elderly RA patients with those of elderly onset RA (EORA)patients. For
example, the author described “Elderly patients with RA seem to have a
characteristic pattern with a more acute onset, systemic involvement,
and worse functional outcomes” (P19, L14-15), but this report compared
EORA patients with young onset RA patients. The reviewer suggests
analyzing the data by stratifying onset age or focusing those in
patients having onset age over 60 years old.

We appreciate your critical comments. According to your important comments, we analyzed the same analysis subjected RA patients with the onset age over 60 years old. We presented these new results in the revised manuscript.

#2 This study applied the 1987 ACR classification, but not 2010 ACR/
EULAR, in spite of the study duration from 2014 to 2021. The reviewer is
afraid that the inclusion criteria missed some of elderly onset RA
patients. The reviewer suggests adding the data when the patients are
enrolled based on the 2010 ACR/EULAR criteria.

We appreciate your critical comments. According to your comments, we applied the 2010 ACR/EULAR classification criteria, in the revised manuscript. However, we could not expand the number of the elderly RA patients.

Minor comments
#3 The reviewer suggests showing the administration routes and the
standard dose of ABT and TCZ, because the standard dose of TCZ is
different among countries. In addition, the interval of subcutaneous
injection with TCZ can be shortened to one week. The reviewer is also
interested in how often the interval is shortened because of the
insufficient efficacy.

We appreciate your critical comments. According to your comments, the administration ruts of ABT and TCZ in the revised manuscript.

#4 Table 1 shows 25.5% of patients treated with ABT and 31.3% of those
with TCZ have received prior Bdmard. The reviewer is afraid it is hard to
interpret the data in patients who had a switch from ABT to TCZ or TCZ
to ABT. To avoid the complexity, the reviewer suggests showing the data
in patients who received ABT or TCZ as the first bDMARD.

We appreciated your critical comments. According to your important comments, we added the important information concerning the prior Biologics in the revised Table 1.

#5 spelling error
“the ACPA possibilities” (P17, L15) => positivity

According to your precise comments, we corrected these mistakes in the revised manuscript.
